# SARS-CoV-2 and its infected world

**DOI:** 10.1093/jmcb/mjaa078

**Published:** 2021-01-25

SARS-CoV-2 is a kind of ‘smart’ virus that generates complex and dynamic crosstalk with hosts. Even with thousands of publications, the researchers have only uncovered the ‘iceberg’ of the virus-infectious properties as well as its effects on human beings, and the approaches against the virus and the related pandemic are limited. In this special issue, several latest publications provide new insights into the relationship between SARS-CoV-2 and its hosts and also deliver potential methods to fight the infectious disease.

In this issue, Dr Jiu and colleagues reviewed the roles of host cytoskeleton such as actin filaments, microtubules, and intermediate filaments in coronavirus infection and concluded that host cytoskeleton homeostasis and modification state are disrupted during coronavirus infection, which is tightly connected to pathological processes. Recently, Dr Zeng and colleagues systematically constructed the human tissue-specific networks associated with SARS-CoV-2 infection, based on collected proteomic datasets from human lung, colon, kidney, liver, and heart. The results showed differentiated interactions between the virus and proteins of diverse tissues and identified key hubs that were regulated by the virus. Importantly, they found that the perturbation of SARS-CoV-2 to the host cell was much smaller than SARS-CoV, which would make the treatment more complicated and uncertain. Dr Schmidtchen and colleagues deeply analyzed the interaction between SARS-CoV-2 spike (S) protein and lipopolysaccharide (LPS) in the blood and indicated that S protein modulated the aggregation state of LPS. The authors found that such interaction resulted in excessive inflammatory response compared with either S protein or LPS alone, suggesting a potential molecular mechanism for the predisposition to SARS-CoV-2 infection in metabolic syndrome patients or others with high levels of LPS in the blood. This work has been highlighted by Drs Carissimo and Ng in this issue.

In addition to LPS, hyperglycemia can significantly increase immune dysfunction of COVID-19. The perspective essay from Dr Meng’s group indicated that diabetic patients were highly susceptible to SARS-CoV-2 infection and COVID-19-associated mortality, suggesting that medications for lowering blood glucose levels or suppressing lactate levels could be beneficial for effective therapeutic treatments for COVID-19 in diabetic patients. Another perspective essay from Drs Yang and Hou’s groups focused on SARS-CoV-2 infection-related anti-viral innate immune response involving pattern-recognition receptors, such as toll-like receptors, retinoic acid-inducible gene I-like receptors, and cyclic guanosine monophosphate‒adenosine monophosphate synthase. The authors further discussed several anti-viral drugs for the treatment of SARS-CoV-2 infection, including type I interferon, chloroquine, and redemsivir.

Although there are no effective medications to cure COVID-19 so far, neutralizing antibodies (NAbs) for blocking SARS-CoV-2 infection are recognized as a promising therapeutic approach. Drs Meng and Yeap’s groups reviewed the technologies applied for SARS-CoV-2 NAb development, as well as common features of these antibodies. Also in this issue, Dr Ryo and colleagues reported a rapid quantitative screening assay for SARS-CoV-2 NAbs based on HiBiT-tagged virus-like particles, greatly accelerating the discovery of SARS-CoV-2 NAbs.

